# Effects of Dietary Supplementation with Black Soldier Fly Larvae (*Hermetia illucens*) Frass on Common Carp (*Cyprinus carpio*)

**DOI:** 10.3390/ani16040693

**Published:** 2026-02-23

**Authors:** Sadia Sultana, Omeralfaroug Ali, Janka Biró, András Szabó, László Ardó, Anita Szűcs, Tamás Gura, Vannaphar Tammajedy, Csaba Hancz, Edward Agyarko, Balázs Kucska

**Affiliations:** 1 Department of Fisheries Research and Development, Institute of Aquaculture and Environmental Safety, Hungarian University of Agriculture and Life Sciences, Guba S. u. 40., 7400 Kaposvár, Hungarykucska.balazs@uni-mate.hu (B.K.); 2 Agribiotechnology and Precision Breeding for Food Security National Laboratory, Department of Physiology and Animal Health, Institute of Physiology and Nutrition, Hungarian University of Agriculture and Life Sciences, Guba S. u. 40., 7400 Kaposvár, Hungary; 3 Research Center for Fisheries and Aquaculture, Institute of Aquaculture and Environmental Safety, Hungarian University of Agriculture and Life Sciences, Anna-Liget. u. 35., 5540 Szarvas, Hungary; 4 HUN-REN-MATE Mycotoxins in the Food Chain Research Group, Guba Sándor u. 40., 7400 Kaposvár, Hungary; 5 Department of Farm Animal Nutrition, Institute of Physiology and Nutrition, Hungarian University of Agriculture and Life Sciences, Guba S. u. 40., 7400 Kaposvár, Hungary

**Keywords:** common carp, larval frass, growth performance, serum biochemical parameters, fatty acid composition of hepatopancreas

## Abstract

Frass is the by-product of the larval meal industry and consists of leftover feed materials, exoskeleton shedding, and larval excrement of black soldier fly larvae (BSFL). To assess the impact of dietary frass (BSFLF) on the growth, feed consumption, biochemical indices, whole-body proximate composition, serum biochemical indices, and fatty acid composition (hepatopancreas) of common carp (*Cyprinus carpio*), an 8-week study was carried out. Juveniles were fed diets with different inclusion of BSFLF levels (0%, 10%, and 20%) in a recirculating aquaculture system, with each diet randomly assigned to a triplicate group of 90 fish (10 fish per tank), with an initial weight of 119.35 ± 30.97 g stocked into 250 L tanks. The study found that increasing dietary frass led to increased growth rates, relative growth rate, weight gain, and protein efficiency ratios, and to decreased feed conversion ratios. Within both total phospholipid and triglyceride fatty acid compositions, the 20% dietary frass increased proportions of saturated and omega-3 fatty acids while decreased the oleic acid (C18:1n9) proportion. To sum up, the use of BSFLF, notably at a 20% inclusion level, as a source of dietary protein has the potential to significantly improve the overall productivity of common carp.

## Simple Summary

Finding alternative feed ingredients is crucial to minimizing reliance on limited traditional resources, despite rising global demand for fish feed. Black soldier fly larvae (BSFL) frass—the excrement and residual feed components from larvae farming—is considered a nutrient-rich, sustainable, and cost-effective ingredient for aquaculture. Our investigation evaluated the impact of black soldier fly larvae frass at inclusion levels of 10% and 20% on the growth performance and physiological development of common carp. Our results suggest that the dietary group incorporating 20% frass can significantly enhance the overall productivity of common carp compared to both the control and the 10% frass groups. In general, frass has the potential to promote the development of sustainable aquaculture diets.

## 1. Introduction

Aquaculture is a rapidly developing sector of animal food production; the apparent consumption of aquatic animal foods has grown dramatically throughout the decades, outpacing the worldwide population’s yearly growth rate [1]. Currently, the sustainability of the aquaculture sector is threatened by high feed costs and excessive reliance on fish meals, prompting fish nutritionists to explore alternative protein sources. Moreover, the aquaculture industry faces the challenge of finding sustainable and affordable substitutes for fish oil and meal, which are the main costly ingredients of current aqua feeds [2].

In recent years, insects have attracted significant interest as alternative sources of protein for humans and livestock, including fish. Insect-derived protein meals could provide a more sustainable option for use in aquaculture compared to conventional protein sources like plant-based sources or fish protein meals [3,4,5]. Animal-derived proteins offer nutritional advantages over plant-based alternatives for fish meal (FM) replacements [6,7,8], with insects being a promising source for alleviating food safety concerns [9,10]. Additionally, the use of insect biomass appears to be more environmentally friendly than conventional animal protein sources. It offers a number of benefits over crop-based ingredients, such as the capacity to be raised on organic waste with little water input, high feed conversion efficiency, reduced greenhouse gas emissions, and minimal land use and water pollution [11].

Frass, a by-product of black soldier fly (*Hermetia illucens*) larvae (BSFL) rearing, has been discovered to be a sustainable substitute feed in the aquaculture sector. It is produced in significant quantities during larval rearing [12]. Its production in the European Union was estimated to reach approximately 1.5 million tons by the mid-2020s [13]. As frass is a by-product, its total production volume is difficult to estimate accurately. However, Gligorescu et al. [14] reported that approximately 1400 kg of former foodstuffs (fresh weight) were required to produce 239 kg of BSFL biomass and 230 kg of frass, corresponding to an almost 1:1 ratio between larval biomass and frass. The quantity of frass is expected to increase further in response to the growing demand for organic waste recycling and policies promoting the circular economy, highlighting the need for research into the sustainable utilization of this by-product [15].

Frass usually has a high mineral content and other beneficial compounds for fish, such as chitin, particularly if the substrate used to cultivate the insects is highly nutritious [16,17]. Recently, it was shown that larval frass has a growth-promoting effect on channel catfish (*Ictalurus punctatus*) [18,19]; Florida pompano (*Trachinotus carolinus*) [20], and hybrid tilapia (*Oreochromis niloticus* × *O. mozambique*) [21] by increasing palatability and feed intake. Furthermore, frass is rich in beneficial microbes [22] nutrients and chitin (a naturally occurring biopolymer from invertebrate exoskeleton), which may function as a prebiotic [20,23,24] by favoring autochthonous bacteria that may prevent harmful bacteria in the digestive tract. Thus, this process can help preserve animal welfare in the fish farming industry.

The common carp (*Cyprinus carpio*) belongs to the family *Cyprinidae* and is a popular, widely produced aquaculture species with high economic value [2]. In 2022, freshwater fishes represented 44 percent of the total finfish and 33 percent of the total aquatic animal production. Carps, barbels, and other cyprinids represented the main group of species produced in 2022, accounting for 18 percent of total aquatic animal production [1]. Common carp contributes up to 8.6% (over 4.2 million metric tons) of the world’s yearly aquaculture production [25], and it also has strong adaptability to dietary changes [26]. Finding sustainable and cost-effective substitutes for the traditional ingredients used in existing aqua feeds is an issue facing the overall aquaculture industry; meanwhile, alternative protein sources for feed need to be investigated [27]. In this regard, research on the use of BSFLF as an alternative ingredient in the aquaculture industry is limited, and a complete lack of research on its effects on common carp necessitates the present study.

Thus, driven by the species importance and to lessen dependency on conventional feedstuffs, the present study aimed to assess the impact of incorporating frass at varying concentrations in the diet on growth performance, feed utilization, selected physiological parameters (including serum biochemistry, biological body indices, and whole body proximate composition), and fatty acid profile of the hepatopancreas in common carp.

## 2. Materials and Methods

### 2.1. Ethical Statement

The present study was reviewed and approved by the ethics committee of the institution of Animal Care and Use, Research Institute of Aquaculture and Fisheries (license number—MATE KC MÁB 2025/1/2). All the required measures, including giving anesthesia and skipping meals before and after measurements, were implemented to reduce the fish’s suffering.

### 2.2. Experimental Fish and Husbandry Conditions

The eight-week experiment was conducted at the Department of Applied Fish Biology, Hungarian University of Agriculture and Life Sciences, Kaposvár Campus, Hungary. Collected fish (1-year-old common carp) were raised in a fish pond (V95 Ltd., Nagyatád, Hungary) and then kept in a recirculating tank under laboratory conditions for 4 weeks for quarantine and adaptation, during which a commercial diet was fed to satiation. After the adaptation period, an entirely randomized design was employed in the study. The fish were placed in 250 L tanks in an experimental recirculating aquaculture system (RAS), which comprised a radial flow settler sedimentation tank (Vidraplast Ltd., Túrkeve, Hungary), a drum filter (Trome DF30, Wolvertem, Belgium), and a moving bed biofilter (MBB), all connected to a 2000-L sump serving as the water recirculation reservoir. The water exchange rate in the culture tanks was 2.5 volumes per hour. Dissolved oxygen concentrations were maintained using air diffusers supplying 10 L min^−1^, manually regulated. Water temperature was controlled indirectly via room air conditioning (Cascade GWH12, Guangdong, China).

The experimental fish (*n* = 90) (119.35 ± 30.97 g weight and 15.90 ± 1.58 cm length) were distributed randomly into three groups (Frass 0%, Frass 10%, and Frass 20%) in triplicate (10 fish per tank) and again acclimatized for one week before the nutritional trial. The fish were fed manually three times a day at 3% bodyweight. Every day after feeding in the afternoon, approximately 15% of the water was removed, and the water was replenished with dechlorinated water. During the feeding trial, the mean pH ranged from 6.5 to 7.5, the dissolved oxygen concentration ranged from 5.8 to 6.3 mg/L, and the temperature ranged from 26.0 °C to 27.3 °C.

### 2.3. Experimental Diet

The basal (control) diet was a commercial feed (Haltáp Ltd., Szarvas, Hungary) composed of fishmeal, wheat, meat meal, extracted soybean meal, corn gluten, hydrolyzed protein, feed fat, pork rind flour, and a vitamin–mineral premix. Two experimental diets were formulated by incorporating black soldier fly larval frass (BSFLF) at inclusion levels of 10% and 20%. These diets were obtained by replacing a mixture consisting of equal parts wheat and extracted sunflower meal, resulting in nearly isonitrogenous and isoenergetic diets (Table 1). Diets were prepared manually by mixing dried ingredients with oil and warm water, using carboxymethyl cellulose as a binder, pelleted, and dried (60 °C) for 48 h until the moisture content was less than 10%. The dried BSFLF was obtained from Agroloop Ltd., Üllő, Hungary. The control diet consisted solely of the basal diet without BSFLF. Fatty acid composition of BSFLF meal and the diets are presented in (Table 2).

### 2.4. Sampling

After 8 weeks of the feeding trial, the fish in each tank were fasted for 24 h and anesthetized with an overdose of clove oil (15 drops per liter natural clove oil (Aromax, Budapest, Hungary)) and weighed to calculate weight gain (WG), specific growth rate (SGR), feed conversion ratio (FCR), survival and protein efficiency ratio (PER). Three randomly collected fish from each tank were weighed, and the total length was measured. These fish were dissected for somatic index measurement. Furthermore, 18 fish (2 individuals per tank, 6 per treatment) were randomly taken and sent to the Hungarian University of Agriculture and Life Sciences, Central Laboratory Department of Food and Feed Safety, for whole-body proximate composition analysis. Before proximate analysis, all of the samples were stored at −20 °C in a refrigerator. The proximate composition of the frass feeds and whole-body (Table 1) was determined using standard techniques of the AOAC [28]. Protein analysis was performed using the Kjeldahl method (AOAC 928.08) using a digestion block (KJELDATHERM, Gerhardt, Königswinter, Germany) via a distillation procedure (VAPODEST 450, Gerhardt, Königswinter, Germany). The crude fat was determined from a 5 g dry sample using the AOAC 945.16 Soxhlet method (SOXTHERM^®^ Unit SOX416, Gerhardt, Königswinter, Germany) and diethyl ether (boiling point, 40–60 °C) as a solvent [28]. The crude ash content was estimated according to the AOAC 942.05 method. Ash was calculated by measuring 2 g of powdered, homogenized samples in a crucible, burning them in a muffle furnace at 550 °C for 3 h, and determining the moisture content in an oven at 105 °C for 4 h. The hepatopancreas was collected from a total of 45 fish (15 per treatment) and stored at −20 °C until fatty acid profile measurements.

### 2.5. Growth Performance Analysis

Mean weight gain was calculated according to Adikwu [29], the relative growth rate was calculated based on Wannigama et al. [30], the specific growth rate was determined according to Auta et al. [31], the protein efficiency ratio followed Abdel-Tawwab [32], and the survival rate was calculated according to Duman [33]. The hepatosomatic index, viscerosomatic index, and condition factor were calculated according to Chemello et al. [34].

Mean Weight Gain (g) (MWG, expressed in grams) = Wt_2_ − Wt_1_

where

Wt_1_ = initial mean weight of the fish at the beginning of the experiment time
Wt_2_ = final mean weight of the fish at the end of the experiment.

$$\mathrm{Feed}\;\mathrm{conversion}\;\mathrm{ratio}\;(\mathrm{FCR})=\frac{\mathrm{weight of feed given}\;(g)}{\mathrm{Fish weight gain}\;(g)}$$

Relative growth rate (RGR, expressed in %) = (W_f_ − W_i_) × 100/W_i_

where

W_i_ = initial average weight at the beginning of the experiment
W_f_ = final average weight at the end of the experiment.

$$\mathrm{Specific}\;\mathrm{growth}\;\mathrm{rate}\;(\mathrm{SGR}\;\%)=100\times \frac{ln\;Wf-ln\;Wi}{t}$$

where W_i_ = initial average weight at the beginning of the experiment, W_f_ = final average weight at the end of the experiment, and t = the number of days for the experiment.

Protein efficiency ratio (PER) = fish weight gain (g)/protein intake (g)

$$\mathrm{Survival}\;\mathrm{rate}\;(\%)=100\times \frac{\mathrm{Number of fish that survived}}{\mathrm{Total number of fish stocked}}$$

Feed intake (FI) (g) = total feed offered over 8 weeks (g)/number of fish in tank

The body condition indices were calculated by the following formulas:

Hepatosomatic index (HSI%) = 100 × liver weight (g)/body weight (g)

Viscera somatic index (VSI%) = 100 × visceral weight (g)/body weight (g)

Condition factor (k) = body weight (g)/total body length (cm) × 100

Nitrogen-free extract (NFE) was calculated as; NFE = 100% − (crude protein % + crude fat % + crude fiber % + crude ash %). Meanwhile, the gross energy was calculated using the following factors according to Schulz et al. [35]: Gross Energy (KJ/g) = 23.9 kJ/g × crude protein (g) + 39.8 kJ/g × crude fat (g) + 17.6 kJ/g × NFE (g) [35].

### 2.6. Biochemical Analysis

At the end of the feeding trial, 6 fish in each experimental group (18 in total, 2 fish per tank) were collected, anesthetized with 15 drops of natural clove oil (Aromax, Hungary) per liter, and weighed individually for blood sampling by puncture of the caudal vein. Serum samples were stored at −20 °C, defrosted at room temperature, and stirred to homogenize using a vortex mixer at 1800 revolutions per minute. Then, 70 µL of the samples were pipetted by Finnpipette^®^ F2 and injected into the cartridge’s specimen inlet (Comprehensive plus 17v). Then, serum levels were measured using a Samsung PT10V blood analyzer and Comprehensive Plus test assays. Total protein concentration was measured using a colorimetric assay and a protein diagnostic reagent kit (FLUITEST TP, Analyticon Biotechnologies AG, Lichtenfels, Germany).

### 2.7. Lipid Composition Analysis

From each treatment, 15 hepatopancreas samples were randomly collected and homogenized (IKA T25 Digital Ultra Turrax, Staufen, Germany) in a 20-fold volume of chloroform/methanol (2:1 *v*/*v*), on an individual basis. The complex lipid content was extracted according to the Folch et al. [36] method. Solvents used in the analysis were ultrapure-grade (Sigma-Aldrich, St. Louis, MO, USA), with 0.01% *w*/*v* butylated hydroxytoluene added to prevent fatty acid oxidation. To separate the lipid fractions of triglycerides and phospholipids, the total lipids that were extracted were put into glass chromatographic columns that held 300 mg of silica gel (230–400 mesh) for every 10 mg of total lipids [37]. Neutral lipids were eluted with 10 mL chloroform, then 15 mL acetone:methanol (9:1, *v*/*v*) was added, while 10 mL pure methanol eluted the total phospholipids.

These extracted fractions were evaporated to dryness under a nitrogen stream and then transmethylated using a base-catalyzed NaOCH_3_ method [38]. Fatty acid methyl esters were extracted into 300 μL ultrapure n-hexane for gas chromatography (AOC 20i automatic injector; Shimadzu 2030, Kyoto, Japan) equipped with a Phenomenex Zebron ZB-WAX plus capillary GC column (30 m × 0.25 mm ID, 0.25 μm film, Phenomenex Inc., Torrance, CA, USA) and a flame ionization detector. Operating conditions included an injector temperature of 220 °C, a detector temperature of 250 °C, and a helium flow rate of 28 cm/s. The oven temperature was programmed as follows: starting from 60 °C with a 2 min hold, increasing to 150 °C, then from 150 to 180 °C at a rate of 2 °C/min with a 10 min hold at 180 °C, and finally from 180 to 220 °C at a rate of 2 °C/min with a 16 min hold at 220 °C (total duration: 74 min). Nitrogen was used as the makeup gas. The calculation was performed with LabSolutions 5.93 software (Shimadzu, Kyoto, Japan) using the Post Run module, with manual peak integration. The identification of fatty acids was performed based on the retention time of a CRM external standard (Supelco 37 Component FAME Mix, Merck-Sigma Aldrich (Darmstadt, Germany), CRM 47885). C22:4n6 and C22:5n6 standards were purchased from Merck-Sigma Aldrich (Darmstadt, Germany) (cat. no.: D3534) and Larodan (Solna, Sweden, cat. no.: 10-2265-4), respectively. Fatty acid results were expressed as the weight percentage of total fatty acid methyl esters.

### 2.8. Statistical Analysis

All datasets were subjected to the Shapiro–Wilk test of normality and the Levene test of equality of variances at the 95% confidence interval level, with p-values ≤ 0.05 to identify parametric and nonparametric variables. A one-way analysis of variance (ANOVA) with a Tukey post hoc test to identify inter-group differences was conducted in R-4.3.2 using the ggplot2 package to create boxplots, whereas the Kruskal–Wallis test was employed within the same software for nonparametric variables. Identified intergroup differences were considered significant when p-values were ≤0.05. Regarding the fatty acid dataset, sparse partial least squares-discriminant analysis (sPLS-DA) was used for dimension reduction and variable selection, yielding the highest classification accuracy [39].

## 3. Results

### 3.1. Growth Parameters and Biological Indices of Common Carp

**Figure 1:**
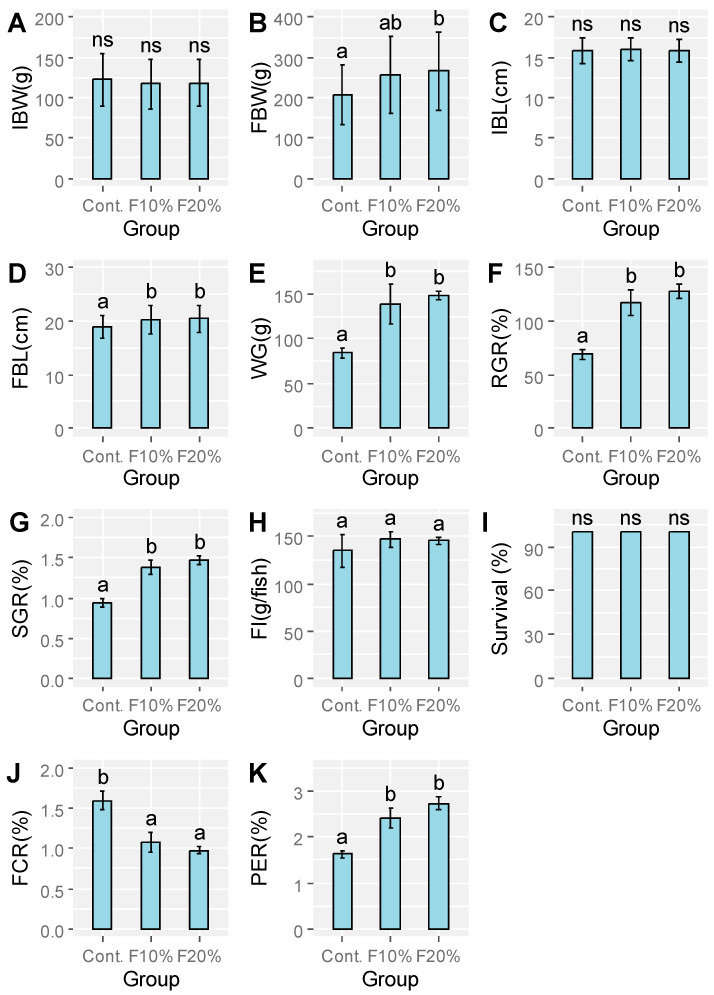
Measurement of IBW or initial body weight (**A**), FBW or final body weight (**B**), IBL or initial body length (**C**), FBL or final body length (**D**), WG or weight gain (**E**), RGR or relative growth ratio (**F**), SGR or specific growth rate (**G**), FI or feed intake (**H**), Survival (**I**), FCR or feed conversion ratio (**J**) and PER or protein efficiency ratio (**K**) of common carp fed the following experimental diets: 0% of BSFLF meal control, 10% of BSFLF (F10%) and 20% of BSFLF (F20%). The data are expressed as the means ± standard deviations, and the use of different letters denotes statistically significant differences between groups (*p* < 0.05), while error lines above bars represent 95% confidence intervals. The symbol “ns” is used to indicate the absence of significant differences (*p* > 0.05).

Growth performance, feed utilization rate, and biological indicators of common carp at different BSFLF inclusion levels are shown in Figure 1 and Table 3. Fish fed with a 20% BSFLF diet had slightly greater growth than those fed with the other feeds. Values of final weight, final length, WG, RGR, SGR, and PER of the carp significantly increased (*p* < 0.05) in groups fed with frass, while FCR values decreased in BSFLF groups compared to the control, indicating improved feed utilization in common carp fed with the BSFLF diet. Biological indices such as the hepatosomatic index (HSI), visceral-somatic index (VSI), gonadosomatic index, and condition factor (k), were not significantly different (*p* > 0.05) among treatments. Throughout the experiment, no common carp mortality was observed, resulting in a 100% survival rate across all treatments.

### 3.2. Whole Body Proximate Composition

The whole-body proximate composition on a wet-weight basis is presented in Table 4. There were no significant differences in the values of these parameters among the fish fed diets containing various levels of larval frass.

### 3.3. Serum Biochemical Parameters

Serum levels of total protein, globulin, glucose, cholesterol, alkaline phosphatase (ALP), amylase, calcium, phosphorus, and albumin/globulin (A/G) ratio were not significantly different (*p* > 0.05) from the dietary frass levels (Table 5). Although serum ALT, lipase, and albumin levels were also measured, their concentrations were below the detection limit and were therefore not considered to indicate adverse effects (Supplementary Table S1).

### 3.4. Fatty Acid Composition of the Hepatopancreas

#### 3.4.1. Fatty Acid Composition of the Hepatopancreas Total Phospholipids

**Figure 2:**
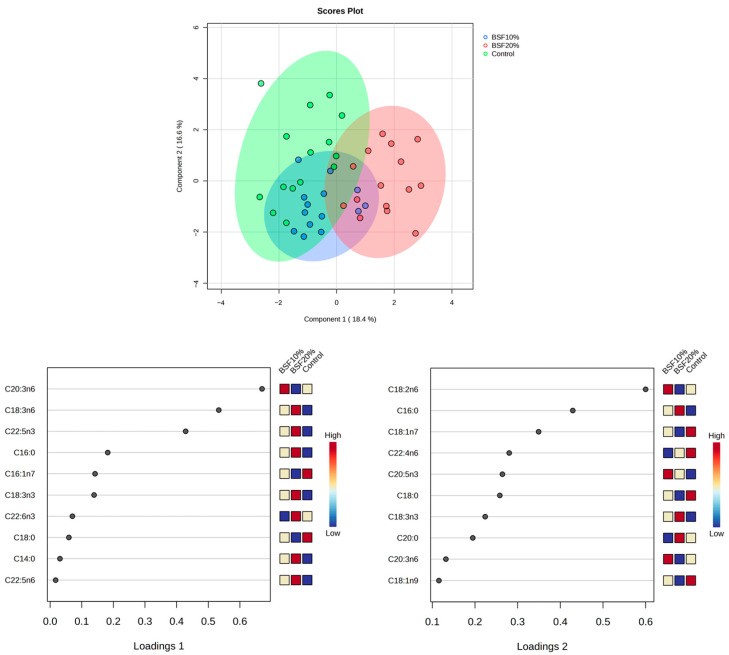
sPLS-DA score plot of the group classification and loadings on the basis of the fatty acid dataset of hepatopancreas phospholipids. Loading values of the 1st and 2nd variates of the lipid dataset based on the sPLS-DA analysis indicate the fatty acids with the highest contribution to classification.

Total phospholipid fatty acid composition in the hepatopancreas of fish fed experimental diets with different BSFLF inclusion rates (0%, 10%, and 20%) is presented in Table 6. Among the saturated fatty acids (C14:0, C16:0, C18:0, and C20:0), proportions of myristic acid (C14:0) and palmitic acid (C16:0) were significantly higher (*p* < 0.05) in the 10% and 20% BSFLF groups than in the control group. In contrast, the stearic acid (C18:0) proportion was significantly lower in BSFL groups. Monounsaturated fatty acids also exhibited proportional differences (decreased in BSFLF 20%), namely in palmitoleic (C16:1n7) and oleic acid (C18:1n9), but their total sums remained insignificant across the different groups. The overall level of omega-3 (n3) increased in the group fed 20% BSFLF, alongside α-linolenic acid (C18:3n3, ALA), docosapentaenoic acid (C22:5n3, or DPA-n3), and docosahexaenoic acid (C22:6n3, or DHA). Similarly, certain omega-6 (n6) fatty acid proportions were increased in the group of BSFLF 20%, such as γ-linolenic acid (C18:3n6, or GLA) and docosapentaenoic acid (C22:5n6, or DPA-n6); however, total n6 levels remained similar across treatments. The n6/n3 ratio decreased in the group fed 20% BSFLF. The distribution of fatty acids in the control group clearly differed from that in the BSFLF 20% group, as shown by the sPLS-DA score plot diagram of total phospholipid fatty acid composition of the hepatopancreas of the fish (Figure 2). Eicosatrienoic acid (C20:3n6), GLA, and DPA-n3 were the most significant contributors to variation on the 1st loading, while linoleic acid (C18:2n6) and palmitic acid were in the 2nd loading.

#### 3.4.2. Fatty Acid Composition of Hepatopancreas Total Triglycerides

**Figure 3:**
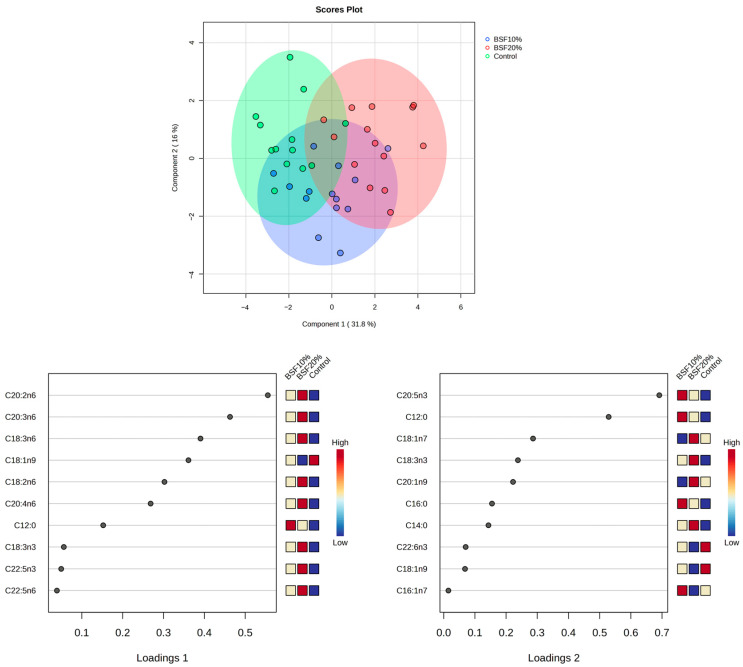
sPLS-DA score plot of the group classification and loading values on the basis of the fatty acid dataset of hepatopancreas triglycerides. Loading values for the 1st and 2nd variates of the lipid dataset, based on the sPLS-DA analysis, indicate the fatty acids with the highest contribution to classification.

Total triglyceride fatty acid compositions of the hepatopancreas of fish fed experimental diets with different BSFLF inclusion rates (0%, 10%, and 20%) are presented in Table 7. Within the saturated fatty acids, lauric acid (C12:0) and myristic acid proportions were significantly higher in the BSFLF groups as compared to the control group. The proportion of oleic acid decreased in the group of BSFLF 20%, whereas the monosaturation level decreased in both groups fed on BSFLF. All proportions of n6 fatty acids were markedly high in the BSFLF 20% group. Consequently, the total n6 proportion increased within the same group. The total level of n3 fatty acid was not significantly different among treatments, but substantial proportional elevations were observed in ALA and DPA-n3 in the BSFLF 20% group. Within the same group, the calculated indices of n6/n3, polyunsaturation level, and unsaturation index significantly increased (Table 7). However, in a multidimensional approach, the groups did not show marked distinctive patterns, as illustrated by the sPLS-DA score plot of total triglyceride fatty acid composition (Figure 3).

## 4. Discussion

Frass from black soldier fly larvae (BSFL) has been evaluated as a feed ingredient for several fish species, including channel catfish [18,19], Florida pompano [20], and hybrid tilapia [21]; however, comparative data for common carp have been lacking. In the present study, the optimal dietary inclusion level of BSFL frass meal for common carp was 20%, based on weight gain and specific growth rate (SGR). Improved growth performance was associated with the adequate nutritional quality of BSFL frass, although additional physiological mechanisms may also be involved. These findings are consistent with those of Yildirim-Aksoy et al. [18], who reported significantly higher final weights in channel catfish fed 100–300 g/kg BSFL frass compared to lower inclusion levels and control diets. Similarly, hybrid tilapia fed diets containing 10–30% BSFL frass showed enhanced weight gain, attributed to improved feed palatability [21]. Supporting evidence from Romano et al. [19] demonstrated that a 10% inclusion of BSFL frass in channel catfish diets significantly increased final weight, weight gain, and feed intake compared to a frass-free control diet after an 8-week feeding trial. In contrast to our findings, Banaver et al. [20] reported that dietary inclusion of BSFL frass at 6, 12, or 18% in the carnivorous Florida pompano resulted in reduced specific growth rate and increased feed conversion ratio compared with the control group. These contrasting results may be attributed to differences in feeding habits and digestive physiology between omnivorous species (e.g., common carp, catfish, and hybrid tilapia) and carnivorous species (Florida pompano), as well as to variations in the nutritional composition of BSFL frass used across studies. Along with increased growth performance, the feed conversion ratio (FCR) decreased from 1.07 to 0.98 with rising BSFL frass inclusion levels (10–20%), indicating improved feed efficiency. As a key indicator of feed suitability in aquaculture, FCR showed a clear dose-dependent response to BSFL frass incorporation. These results are consistent with previous studies in channel catfish [18] and hybrid tilapia [21], which reported a progressive reduction in FCR with increasing BSFL frass inclusion, with optimal efficiency around 10%. It is noteworthy that survival rates remained consistent across all dietary treatments examined in the current investigation indicates positive impact of frass meal on the survival of common carp, aligning with previous studies on channel catfish [18,19] and hybrid tilapia [21]. Furthermore, the inclusion of 10 or 20% frass in the dietary composition resulted in significantly higher protein utilization compared to the control dietary treatment. The enhanced protein accessibility from frass compared to plant-derived sources may be attributed to the prevalence of antinutritional factors commonly present in plant feed ingredients, which can compromise nutrient bioavailability [40]. However, additional investigations are warranted to elucidate the underlying digestibility processes. Furthermore, several studies have shown that incorporating BSFL frass as a nutritional additive improves specific growth rate and feed conversion efficiency in channel catfish and hybrid tilapia, particularly when incorporation levels are kept below 20% [19,41]. Furthermore, the presence of antimicrobial peptides and chitinous substances within black soldier fly larvae frass may have contributed to the enhanced feed utilization efficiency and increased nutrient uptake observed in 10 or 20% BSFLF diet. It is conceivable that higher dosages of BSFLF supplementation may influence gastrointestinal microbial communities, thereby promoting improved digestive efficiency and nutrient absorption capacity, ultimately leading to enhanced growth performance, although other factors may also be involved. The efficiency of nutrient uptake depends on the duration and extent of contact between nutrients and the absorptive epithelial surface. The composition of dietary intake has been demonstrated to influence both digestive enzymatic activity and the transit duration of digesta throughout the gastrointestinal system [42,43]. Non-digestible dietary components are expected to affect nutrient translocation in the gastrointestinal tract, thereby influencing overall nutrient absorption. Consequently, the presence of inorganic mineral compounds and chitin may enhance intestinal bulk formation, decrease fecal retention duration, and reduce enzymatic accessibility to target substrates.

No significant differences were observed among dietary treatments in the hepatosomatic index (HSI) and viscerosomatic index (VSI) of common carp (Table 3), consistent with previous studies in tilapia [21] and channel catfish [18]. Similarly, channel catfish fed diets containing 5–30% BSFL frass showed no significant changes in biological indices [18]. Since alterations in HSI are generally associated with hepatic dysfunction [44], the comparable values observed across treatments suggest that frass inclusion did not impair liver health. Although fish fed 20% frass exhibited slightly lower HSI (1.88) and those fed 10% frass showed slightly higher values (2.22), these differences were not statistically significant. Variability in HSI reported elsewhere may relate to differences in frass composition, which depends on the larval rearing substrate and may influence hepatic nutrient deposition [45].

Whole-body proximate composition was not affected by dietary frass inclusion, in agreement with findings in hybrid tilapia [21] and channel catfish [18], where up to 30% BSFL frass did not alter body composition. These results indicate that frass supplementation does not modify overall somatic composition, even when growth performance is improved at higher inclusion levels.

Hematological and serum biochemical parameters, important indicators of physiological stress and health status [46], also remained unaffected by dietary treatments. Elevated ALP and ALT levels are typically associated with hepatic dysfunction [47,48,49]; however, no significant differences were detected in total protein, globulin, glucose, cholesterol, ALP, amylase, calcium, or phosphorus. These findings agree with Yildirim-Aksoy et al. [50], who reported no significant changes in serum biochemical indices of channel catfish fed 10–30% BSFL frass, and with studies in hybrid tilapia showing no adverse hepatic effects at inclusion levels up to 30% [21]. A slight increase in total protein concentration (2.87 to 3.03 g/dL) with increasing frass inclusion was observed, similar to previous findings in catfish [50]. Overall, the results indicate that dietary BSFL frass had no detrimental effects on liver function, cellular integrity, or oxygen transport capacity [51], supporting the observed growth performance outcomes.

Dietary fatty acid composition had an effect on the fatty acid composition of several fish that was revealed in some earlier studies [52,53,54]. In the present study, increasing levels of BSFLF led to a significant increase in saturated fatty acids (myristic and palmitic acids), GLA, DPA-n6, ALA, DPA-n3, and DHA in the hepatopancreas membrane of common carp (Table 6). However, the proportions of certain monounsaturated fatty acids (notably palmitoleic and oleic acids) were decreased. It is worth mentioning that the literature lacks data on hepatopancreas membrane lipid composition in relation to dietary BSFL or BSFLF. It is evident that the BSFLF fatty acid composition does not markedly modify the dietary composition, at least up to a 20% inclusion level. Thus, the elevated proportions in ALA (an essential fatty acid) and other polyunsaturated fatty acids suggest either high dietary intake or modulations within lipid metabolism processes (biosynthesis and incorporation). Both scenarios appear plausible since BSFLF is associated with improved palatability [50], and DPA-n3/n6 and DHA proportions increased in both phospholipid and triglyceride fractions, whereby the production of these fatty acids is driven by high C24:6n3 production and incorporation rate into mitochondrial β-oxidation, as well as elevated Δ4 and Δ6 desaturase activities [55]. Furthermore, the marked proportional increases in lauric acid, myristic acid, and n6 fatty acids detected in hepatopancreas triglycerides support these scenarios. These findings are also in accordance with those of earlier studies on common carp [56], Jian carp [57], and rainbow trout [58] fed diets with different inclusion levels of insect meal and BSFL.

Fish fed BSFL frass diets provided a high proportion of the myristic acid in both polar and unipolar lipid fractions, alongside the elevation of lauric acid proportion in triglycerides. According to Romano et al. [19], among the fatty acids, fish fed 10% BSFL diets had noticeably higher levels of myristic acid and lauric acid, which are generally rich in BSFL meal and oil, and possess antimicrobial and antiviral qualities [59,60]. However, lauric acid was not a predominant fatty acid in BSFLF, highlighting lipid compositional differences between BSFLF and BSFL meals, a novelty of this study. Notably, lauric acid was not detected in the hepatopancreas membrane of common carp but was detected in hepatopancreas triglycerides in those fed diets containing BSFL frass, supporting the proposal of modulation in lipid metabolism towards the production of medium- and long-chain fatty acids as demonstrated by the decreased stearic acid proportion. Nevertheless, additional research on the potential role of BSFLF in fish nutrition should be conducted, with attention to its nutritive value relative to BSFL and its effects on metabolic processes in different fish species.

In the present study, n6/n3 of fish hepatopancreas membranes from the BSFLF 20% group markedly decreased (2.40 compared to 2.85 in the control), whereas the hepatopancreas triglyceride level significantly increased in the hepatopancreas of fish fed the highest level of BSFLF. The frequency of chronic food-related disorders can be decreased by keeping the n6/n3 ratio below 4, as per human health recommendations [61]. In the hepatopancreas membrane, the polyunsaturation level did not differ among groups, whereas it increased with the inclusion of 10% and 20% dietary frass in hepatopancreas triglycerides. The ratio of polyunsaturated fatty acids to saturated fatty acids in animal products should be greater than 0.4 to lower the risk of autoimmune, cardiovascular, and other chronic illnesses [62]. In the present study, dietary frass had no discernible impact on this ratio, which is remarkably consistent with previous research in which dietary BSFL frass was fed to channel catfish [18,19]. However, the overall proportion of polyunsaturated fatty acids compromised the total monounsaturation level, suggesting potentially distinct biochemical pathways that prioritize the production of polyunsaturated fatty acids.

In summary, our findings indicate the significant promise of integrating black soldier fly larvae frass meal into aquafeed compositions as ecologically sustainable alternative protein ingredients for economically significant cultured fish species. Nonetheless, to corroborate and extend the preliminary findings, subsequent research endeavors ought to incorporate histological evaluations to deepen understanding of subclinical impacts of frass on fish physiological indicators, thereby facilitating the optimization of black soldier fly larvae frass meal integration into aquaculture feed formulations.

## 5. Conclusions

The present study revealed that feeding larval frass to juvenile common carp at up to 20% had a positive effect on growth, survival, and feed conversion efficiency, with no adverse effects on whole-body proximate composition, serum parameters, and fatty acid composition (hepatopancreas). The majority of the body indices, including the condition factor and hepato-somatic index, showed no discernible changes. BSFLF can significantly improve fish production performance (SGR and FCR) and may be an affordable, sustainable feed ingredient for fish diets. Furthermore, future research could explore the nutritional digestibility of BSFLF diets and their immune-stimulatory effect on immune parameters and fish resistance to infectious pathogens. Given the limited scholarly work in this field and the anticipated growth of the insect cultivation industry, understanding the influence of dietary frass on fish health and productivity becomes increasingly significant. Such research endeavors would facilitate the optimization of BSFL-derived products for incorporation into aquaculture feed formulations.

## Figures and Tables

**Table 1:** Formulation of experimental diets (BSFLF 10% and 20%) and control diets for common carp in the nutritional trial.

Ingredients (g/kg)	Control	BSFLF 10%	BSFLF 20%
base feed ^a^	750	750	750
wheat+ ext. sunflower (80% + 20%)	200	100	0
Frass ^b^	0	100	200
oil (poultry)	50	50	50
**Proximate composition (% of dry matter)**			
moisture content	6.9	7.3	7.2
crude protein	35.4	36.9	35.2
crude fat	9.6	9.3	9.7
crude fiber	2.7	3.9	5.1
crude ash	7.3	8.5	9.4
Nitrogen-free energy (NFE)	45	41.5	40.6
Gross Energy (KJ/g)	20.20	19.82	19.42

^a^ Base feed from Haltáp Ltd., Szarvas, Hungary. ^b^ Frass (dried) from a Hungarian insect producer (Agroloop Ltd.).

**Table 2:** Fatty acid composition of black soldier fly larvae frass (BSFLF) meal and the diets (Control—0% BSFLF, BSFLF 10%, and 20%) used in the study.

Fatty Acid	Control	BSFLF 10%	BSFLF 20%	BSFLF as an Ingredient
C12:0	0.07	0.05	0.06	0.00
C14:0	0.70	0.66	0.65	1.21
C16:0	19.6	19.6	19.7	23.0
C18:0	5.81	5.77	5.76	9.03
C20:0	0.19	0.18	0.18	0.74
saturation	26.4	26.2	26.3	34.0
C16:1n7	4.12	4.05	4.12	4.54
C18:1n7	1.64	1.62	1.62	4.10
C18:1n9	31.2	31.5	31.5	29.8
C20:1n9	0.55	0.55	0.52	1.17
monounsaturation	37.5	37.7	37.8	39.6
C18:2n6	31.9	32.0	31.8	24.0
C18:3n6	0.06	0.07	0.07	-
C20:2n6	0.16	0.16	0.16	-
C20:3n6	0.08	0.10	0.10	0.70
C20:4n6	0.67	0.68	0.71	0.99
C22:4n6	0.15	0.13	0.12	-
C22:5n6	0.06	0.05	0.06	-
omega-6	33.1	33.2	33.1	25.7
C18:3n3	1.92	1.87	1.83	0.65
C20:5n3	0.29	0.27	0.22	-
C22:5n3	0.09	0.08	0.08	-
C22:6n3	0.80	0.73	0.72	-
omega-3	3.10	2.95	2.85	0.65
omega-6/omega-3	10.7	11.2	11.6	39.9
polyunsaturation	32.0	32.1	32.0	24.0

Abbreviation: -, not detected.

**Table 3:** Effects of different inclusion levels of frass (BSFLF 0% (control), BSFLF 10%, and BSFLF 20%) on the body condition indices of common carp.

Parameters	Control	BSFLF 10%	BSFLF 20%	*p*-Value
hepatosomatic index (%)	2.09 ± 0.77	2.22 ± 0.45	1.87 ± 0.76	0.54
viscera-somatic index (%)	9.48 ± 2.55	7.32 ± 1.27	7.24 ± 2.17	0.50
gonadosomatic index (%)	3.09 ± 1.41	2.10 ± 1.16	2.76 ± 1.40	0.29
condition factor (k) (g/cm^3^)	2.81 ± 0.36	2.92 ± 0.40	3.05 ± 0.17	0.31

The data are expressed as the means ± standard deviations (*n* = 9 fish/group).

**Table 4:** Effects of different inclusion levels of frass (BSFLF 0% (control), 10%, and 20%) on the proximate composition of the whole body of common carp (% as fed).

Parameters	Control	BSFLF 10%	BSFLF 20%	*p*-Value
moisture (%)	67.9 ± 2.35	69.1 ± 1.04	70.9 ± 2.01	0.225
crude protein (%)	15.4 ± 1.18	15.5 ± 0.17	15.0 ± 0.36	0.672
crude fat (%)	13.8 ± 3.35	10.8 ± 0.76	11.5 ± 2.21	0.348
ash (%)	2.33 ± 0.25	2.33 ± 0.21	2.57 ± 0.12	0.326

The data are expressed as the means ± standard deviations of triplicate samples.

**Table 5:** Serum chemistry values of common carp fed diets (BSFLF—0%, 10%, and 20%) containing different inclusion levels of frass for 8 weeks.

Parameters	Control	BSFLF 10%	BSFLF 20%	*p*-Value
total protein (g/dL)	2.87 ± 0.06	3.30 ± 0.17	3.03 ± 0.38	0.804
globulin (g/dL)	2.17 ± 0.11	2.40 ± 0.10	2.30 ± 0.26	0.168
albumin (g/dL)	0.7 ± 0.1	0.9 ± 0.2	0.7 ± 0.1	0.933
glucose (mg/dL)	127.3 ± 32.6	106.0 ± 18.3	98.0 ± 5.29	0.171
cholesterol (mg/dL)	127.7 ± 12.7	121.0 ± 2.65	122.7 ± 17.7	0.335
alkaline phosphatase (U/L)	87.7 ± 49.9	78.7 ± 30.0	68.3 ± 7.09	0.296
amylase (U/L)	221.0 ± 91.5	246.0 ± 97.9	152.7 ± 37.9	0.518
calcium (mg/dL)	9.30 ± 0.56	9.60 ± 1.42	9.30 ± 1.06	0.791
phosphorus (mg/dL)	5.23 ± 1.63	5.60 ± 0.46	6.27 ± 0.70	0.397
A/G (albumin/globulin)	0.33 ± 0.06	0.37 ± 0.58	0.30 ± 0.00	0.306

**Table 6:** Total phospholipid fatty acid composition of the hepatopancreas of fish fed experimental diets with different BSFLF inclusion rates (0%, 10%, and 20%), with *n* = 15 samples/treatment.

Fatty Acid	Control	BSFLF 10%	BSFLF 20%
C14:0	0.23 ± 0.06 ^b^	0.25 ± 0.05 ^a^	0.29 ± 0.06 ^a^
C16:0	21.9 ± 2.30 ^b^	23.8 ± 1.33 ^a^	24.3 ± 1.15 ^a^
C18:0	13.6 ± 1.45 ^a^	12.4 ± 1.57 ^b^	12.0 ± 0.78 ^b^
C20:0	0.09 ± 0.03	0.08 ± 0.02	0.09 ± 0.01
saturated	35.8 ± 3.04	36.6 ± 1.87	36.7 ± 1.02
C16:1n7	1.19 ± 0.18 ^a^	1.18 ± 0.16 ^b^	1.04 ± 0.12 ^b^
C18:1n7	1.87 ± 0.30	1.68 ± 0.18	1.68 ± 0.17
C18:1n9	13.3 ± 1.62 ^a^	12.3 ± 1.40 ^ab^	11.9 ± 1.06 ^b^
C20:1n9	1.54 ± 0.53	1.37 ± 0.20	1.41 ± 0.24
monounsaturated	17.9 ± 2.27	16.5 ± 1.46	16.0 ± 1.33
C18:2n6	5.88 ± 1.47	6.77 ± 0.78	5.93 ± 0.95
C18:3n6	0.15 ± 0.06 ^b^	0.23 ± 0.09 ^b^	0.31 ± 0.12 ^a^
C20:2n6	1.01 ± 0.30	1.04 ± 0.23	1.06 ± 0.18
C20:3n6	4.25 ± 1.00 ^a^	4.41 ± 0.57 ^a^	2.96 ± 0.47 ^b^
C20:4n6	16.7 ± 2.51	16.0 ± 0.85	16.3 ± 1.34
C22:4n6	1.39 ± 0.40	1.22 ± 0.17	1.32 ± 0.32
C22:5n6	4.59 ± 1.12 ^b^	4.89 ± 0.56 ^ab^	5.36 ± 0.59 ^a^
omega-6	34.0 ± 2.11	34.6 ± 1.39	33.2 ± 0.92
C18:3n3	0.07 ± 0.02 ^b^	0.09 ± 0.02 ^ab^	0.10 ± 0.02 ^a^
C20:5n3	0.27 ± 0.09	0.31 ± 0.05	0.27 ± 0.07
C22:5n3	0.45 ± 0.10 ^b^	0.46 ± 0.07 ^b^	0.60 ± 0.15 ^a^
C22:6n3	11.5 ± 1.66 ^b^	11.5 ± 1.81 ^ab^	13.1 ± 1.91 ^a^
omega-3	12.2 ± 1.72 ^b^	12.4 ± 1.84 ^b^	14.1 ± 1.93 ^a^
omega-6/omega-3	2.82 ± 0.35 ^a^	2.85 ± 0.38 ^a^	2.40 ± 0.30 ^b^
polyunsaturated	46.2 ± 3.39	47.0 ± 2.59	47.3 ± 1.48
unsaturation index	212.8 ± 16.9	212.7 ± 12.3	220.2 ± 10.6
average chain length	18.7 ± 0.15	2.85 ± 0.10	18.7 ± 0.10

The data represent the mean ± standard deviation (SD). ^a,b^, different subscript letters indicate significant differences at *p* < 0.05.

**Table 7:** Total triglyceride fatty acid composition of hepatopancreas of fish fed experimental diets with different BSFLF inclusion rates (0%, 10%, and 20%), with *n* = 15 samples/treatment.

Fatty Acid	Control	BSFLF 10%	BSFLF 20%
C12:0	0.02 ± 0.01 ^b^	0.04 ± 0.00 ^a^	0.04 ± 0.01 ^a^
C14:0	0.92 ± 0.11 ^b^	1.02 ± 0.10 ^a^	1.03 ± 0.08 ^a^
C16:0	18.2 ± 1.26	19.0 ± 1.31	18.3 ± 1.97
C18:0	8.03 ± 2.37	6.66 ± 1.50	6.26 ± 1.67
C20:0	0.17 ± 0.04	0.16 ± 0.04	0.16 ± 0.04
saturated	27.3 ± 3.46	26.8 ± 2.35	25.8 ± 3.25
C16:1n7	5.34 ± 0.64	5.35 ± 0.62	4.86 ± 0.60
C18:1n7	2.50 ± 0.36	2.35 ± 0.15	2.57 ± 0.30
C18:1n9	50.5 ± 3.50 ^a^	46.1 ± 2.81 ^ab^	44.3 ± 3.19 ^b^
C20:1n9	1.86 ± 0.41	1.74 ± 0.19	1.97 ± 0.28
monounsaturated	60.2 ± 4.10 ^a^	55.5 ± 3.21 ^b^	53.7 ± 3.45 ^b^
C18:2n6	10.4 ± 2.55 ^b^	15.0 ± 3.06 ^a^	17.2 ± 5.40 ^a^
C18:3n6	0.20 ± 0.05 ^b^	0.24 ± 0.07 ^ab^	0.41 ± 0.17 ^a^
C20:2n6	0.28 ± 0.08 ^b^	0.35 ± 0.10 ^b^	0.51 ± 0.10 ^a^
C20:3n6	0.33 ± 0.09 ^b^	0.38 ± 0.08 ^b^	0.52 ± 0.10 ^a^
C20:4n6	0.50 ± 0.18 ^b^	0.57 ± 0.14 ^b^	0.81 ± 0.25 ^a^
C22:4n6	0.04 ± 0.03	0.06 ± 0.02	0.07 ± 0.03
C22:5n6	0.06 ± 0.03 ^b^	0.09 ± 0.03 ^ab^	0.10 ± 0.03 ^a^
omega-6	11.8 ± 2.69 ^b^	16.7 ± 3.32 ^a^	19.7 ± 5.37 ^a^
C18:3n3	0.47 ± 0.17 ^b^	0.75 ± 0.17 ^a^	0.76 ± 0.31 ^a^
C20:5n3	0.06 ± 0.03 ^b^	0.10 ± 0.04 ^a^	0.07 ± 0.02 ^b^
C22:5n3	0.02 ± 0.01 ^b^	0.03 ± 0.01 ^ab^	0.03 ± 0.01 ^a^
C22:6n3	0.22 ± 0.33	0.07 ± 0.09	0.06 ± 0.05
omega-3	0.77 ± 0.40	0.95 ± 0.16	0.93 ± 0.33
omega-6/omega-3	17.1 ± 3.97 ^b^	17.7 ± 2.49 ^b^	22.0 ± 2.92 ^a^
polyunsaturated	12.5 ± 2.90 ^b^	17.6 ± 3.44 ^a^	20.6 ± 5.68 ^a^
unsaturation index	88.6 ± 5.31 ^b^	94.3 ± 5.21 ^ab^	99.1 ± 8.89 ^a^
average chain length	17.6 ± 0.04	17.5 ± 0.04	17.6 ± 0.05

The data represent mean ± standard deviation (SD). ^a,b^, different subscript letters indicate significant differences at *p* < 0.05.

## Data Availability

Data available on request due to restrictions, e.g., privacy or ethical reasons.

## Supplementary Materials

The following supporting information can be downloaded at https://www.mdpi.com/article/10.3390/ani16040693/s1. Table S1: Serum chemistry values of common carp fed diets (BSFLF—0%, 10%, and 20%) containing different inclusion levels of frass for 8 weeks.
