# Supplementary material for: Effects of Dietary Supplementation with Black Soldier Fly Larvae (Hermetia illucens) Frass on Common Carp (Cyprinus carpio)
Source: Animals (Basel). 2026 Feb 23;16(4):693. doi: 10.3390/ani16040693 (PMC12937264; doi:10.3390/ani16040693)
Supplement: Supplementary file 1 [file animals-16-00693-s001.zip › animals-3864889-supplementary.pdf]

Supplementary Materials

Table S1. Serum chemistry values of common carp fed diets (BSFLF - 0%, 10%, and 20%) containing different inclusion levels of frass for 8 weeks.

	No. Fish	ALT(T) U/L	ALB(T) g/dl	LIPA U/L
**Control**	1	< 10	< 1.0	< 20
	2	12	1.2	< 20
	3	< 10	1.0	< 20
	4	15	< 1.0	< 20
	5	< 10	1.3	< 20
	6	< 10	1.1	< 20
**BSFLF 10%**	1	< 10	< 1.0	< 20
	2	15	< 1.0	< 20
	3	14	< 1.0	< 20
	4	12	< 1.0	< 20
	5	< 10	1	< 20
	6	< 10	1.1	< 20
**BSFLF 20%**	1	16	< 1.0	< 20
	2	< 10	< 1.0	< 20
	3	14	< 1.0	< 20
	4	< 10	< 1.0	< 20
	5	21	< 1.0	< 20
	6	< 10	< 1.0	< 20
